# LncRNA NEAT1 promotes epithelial–mesenchymal transition in nasal polyp cells via the miR-199-3p/PAK4 axis

**DOI:** 10.3389/fimmu.2025.1613179

**Published:** 2025-06-30

**Authors:** Shuman Li, Yu Jiang, Yalan Zhang, Bowen Zheng, Chao Yuan, Yang Shen, Yi Zhao, Tao Lu, Yucheng Yang

**Affiliations:** ^1^ Department of Otorhinolaryngology, Upper Airway Inflammation and Tumor Laboratory, The First Affiliated Hospital of Chongqing Medical University, Chongqing, China; ^2^ Department of Pediatrics, Chongqing Hospital of Traditional Chinese Medicine, Chongqing, China; ^3^ Chongqing Key Laboratory of Translational Research for Cancer Metastasis and Individualized Treatment, Chongqing University Cancer Hospital, Chongqing, China

**Keywords:** CRSwNP, NEAT1, EMT, miR-199-3p, PAK4, non-coding RNA

## Abstract

**Background and purpose:**

Chronic rhinosinusitis with nasal polyps (CRSwNP) is a persistent inflammatory condition marked by high recurrence and limited therapeutic efficacy. This study investigates the role of long non-coding RNA NEAT1 in promoting epithelial–mesenchymal transition (EMT) in CRSwNP, focusing on its regulatory interaction with the miR-199-3p/PAK4 axis.

**Methods:**

NEAT1 expression was assessed in nasal epithelial cells from CRSwNP patients using qPCR and FISH. Primary human nasal epithelial cells and BEAS-2B cells were subjected to NEAT1 knockdown via siRNA. Cell migration, barrier function, and cytoskeletal dynamics were evaluated through scratch assays, Transwell migration, FITC-Dextran permeability testing, and phalloidin staining. EMT marker expression was analyzed via Western blotting and immunofluorescence. Transcriptome sequencing identified PAK4 as a downstream effector. *In vivo* validation was performed using a mouse nasal polyp model, and molecular interactions among NEAT1, miR-199-3p, and PAK4 were confirmed via dual-luciferase reporter assays. Rescue experiments further elucidated mechanistic pathways.

**Results:**

In comparison to controls, NEAT1 expression was significantly elevated in the epithelial tissues of CRSwNP. NEAT1 knockdown inhibited cell migration, enhanced epithelial barrier integrity, and reversed EMT-associated cytoskeletal remodeling. E-cadherin levels increased, while N-cadherin and vimentin decreased. Transcriptomic and functional analyses identified PAK4 as a NEAT1-regulated target. NEAT1 was shown to sponge miR-199-3p, thereby relieving its inhibitory effect on PAK4. Overexpression of miR-199-3p suppressed PAK4 and mitigated EMT-related changes induced by NEAT1.

**Conclusion:**

NEAT1 promotes EMT in nasal polyp epithelial cells by modulating the miR-199-3p/PAK4 axis, highlighting its potential as a diagnostic biomarker and therapeutic target in CRSwNP.

## 1 Introduction

CRSwNP is a chronic inflammatory disorder characterized by persistent inflammation of the nasal and sinus mucosa and the formation of nasal polyps (1–3). Clinically, CRSwNP presents with symptoms such as nasal obstruction, rhinorrhea, and hyposmia, contributing to substantial morbidity (4–6). Despite advances in medical and surgical therapies, high recurrence rates and limited long-term efficacy underscore the need for deeper insight into its molecular mechanisms (7–9).

Recent studies have highlighted the role of EMT in CRSwNP pathogenesis. EMT is a biological process in which epithelial cells lose polarity and adhesion, acquiring mesenchymal traits such as increased motility (10, 11). This transition contributes to tissue remodeling, epithelial barrier dysfunction, and polyp formation under the influence of a Th2-biased inflammatory environment (12).

Epigenetic regulation, especially through non-coding RNAs (ncRNAs), plays a crucial role in controlling EMT (13–15). Among these, long non-coding RNAs (lncRNAs) have emerged as key regulators of gene expression and cell phenotype (16). NEAT1 (Nuclear Enriched Abundant Transcript 1), a well-characterized lncRNA, has been implicated in inflammation, immune regulation, and EMT in various pathological contexts (17–19), yet its function in CRSwNP remains largely unexplored.

In our preliminary transcriptomic analysis of nasal polyp epithelial cells, we identified PAK4 (p21-activated kinase 4) as a potential downstream effector of NEAT1. PAK4 is a serine/threonine kinase known to modulate cytoskeletal dynamics, cell migration, and EMT (20–22). Its dysregulation is linked to pathological remodeling in several diseases.

Given the growing evidence that lncRNAs can act as competing endogenous RNAs (ceRNAs) to sequester microRNAs and regulate target gene expression, we hypothesized that NEAT1 might promote EMT through a ceRNA mechanism. In this context, we identified miR-199-3p, a microRNA known to suppress EMT and regulate tissue remodeling, as a functional intermediary between NEAT1 and PAK4.

This study aims to elucidate the molecular mechanism by which NEAT1 promotes EMT in CRSwNP via the miR-199-3p/PAK4 axis. By integrating transcriptomic data, *in vitro* cell models, and *in vivo* validation, we provide evidence for a novel regulatory pathway contributing to epithelial dysfunction and polyp development, offering potential targets for precision diagnosis and therapeutic intervention in CRSwNP.

## 2 Materials and methods

### 2.1 Clinical tissue samples

This study included 32 patients diagnosed with chronic rhinosinusitis with nasal polyps who underwent treatment at the Department of Otorhinolaryngology between December 2022 and September 2023. Twelve patients with nasal septum deviation, treated during the same period, were recruited as the control group. Nasal polyp tissues in the CRSwNP group were obtained during endoscopic sinus surgery, while normal nasal mucosa samples in the control group were collected from hypertrophic or bullous middle turbinate mucosa during corrective septal procedures. The institutional ethics committee of the First Affiliated Hospital of Chongqing Medical University granted approval for this study (Approval No.2022-K301).

### 2.2 Cell lines and primary cell isolation

The human bronchial epithelial cell line (BEAS-2B) was obtained from Pronas Biotechnology (China). Primary human nasal epithelial cells (hNECs) were isolated from nasal polyp or middle turbinate mucosa collected during surgery at the Department of Otorhinolaryngology. Tissues were transferred to the laboratory within 30 minutes and processed under sterile conditions to extract epithelial cells using standard enzymatic dissociation protocols. Cells were cultured in Bronchial Epithelial Cell Growth Medium (BEGM) supplemented with necessary growth factors. All cells were maintained at 37°C in a humidified incubator with 5% CO_2_.

In screening tool cells, we selected the immortalized bronchial epithelial cell line BEAS-2B based on the following rationale: (1) Nasal polyp epithelium and bronchial epithelium share pathological similarities, both being pseudostratified ciliated columnar epithelia, allowing BEAS-2B to effectively mimic nasal polyp biology; (2) the hNECs exhibit significant limitations, including poor tolerance to lipofectamine transfection, low transfection efficiency, and high cellular heterogeneity. In contrast, BEAS-2B, due to its immortalized nature, overcomes these technical challenges, providing a stable and reproducible model for functional validation. This selection ensures both pathological relevance and experimental feasibility.

### 2.3 Transwell assays

Cell migration was assessed using Transwell chambers following protocols reported in our prior publication (23). Briefly, cells were seeded into the upper chambers without Matrigel coating, and migrated cells were fixed, stained, and quantified after 24 h.

### 2.4 Wound healing assay

BEAS-2B and primary hNECs were subjected to a wound healing assay as previously described (23). In brief, a scratch was created across a confluent monolayer, and cell migration into the wound area was assessed over 24 hours.

### 2.5 Epithelial barrier permeability assay

The integrity of the epithelial barrier was evaluated using a FITC–dextran (4 kDa; Sigma-Aldrich) translocation assay. Cells were grown on 0.4 μm Transwell filters until a continuous monolayer was established. After rinsing with phosphate-buffered saline (PBS), 500 μL of FITC–dextran solution was applied to the upper (apical) chamber, while 1.5 mL of serum-free medium was added to the lower (basolateral) chamber. Following a 2-hour incubation at 37 °C, 100 μL of the basolateral medium was collected for fluorescence measurement using a microplate reader (excitation at 485 nm, emission at 535 nm). Elevated fluorescence intensity reflected increased paracellular flux, indicative of barrier dysfunction.

### 2.6 Phalloidin staining for F-actin cytoskeleton

To visualize the F-actin cytoskeleton, cells were stained using Actin-Tracker Red-Rhodamine (Phalloidin; Beyotime, China). Cells were cultured on sterile glass coverslips until they reached approximately 75% confluence. After completing the designated treatments, cells were fixed with 4% paraformaldehyde at room temperature for 15 minutes, then permeabilized using 0.1% Triton X-100 for 10 minutes. Subsequently, cells were incubated in Actin-Tracker Red-Rhodamine solution for 30 minutes in the dark to stain F-actin. Nuclear staining was performed with DAPI for 5 minutes. Finally, coverslips were mounted with anti-fade medium, and fluorescence images were acquired using a fluorescence microscope. F-actin distribution and cytoskeletal remodeling were analyzed qualitatively.

### 2.7 Quantitative real-time PCR

Total RNA extraction, cDNA synthesis, and quantitative real-time PCR (qPCR) were performed as previously described in our published study (23). The specific primer sequences used are provided in **Supplementary Table 2**.

### 2.8 Western blot

Western blotting was conducted as previously reported (24), with slight modifications. Briefly, proteins from RIPA lysates were resolved via SDS-PAGE, transferred onto PVDF membranes, and incubated with specific primary antibodies. Antibody information are listed in **Supplementary Table 3**.

### 2.9 Cell transfection

**Figure 1:**
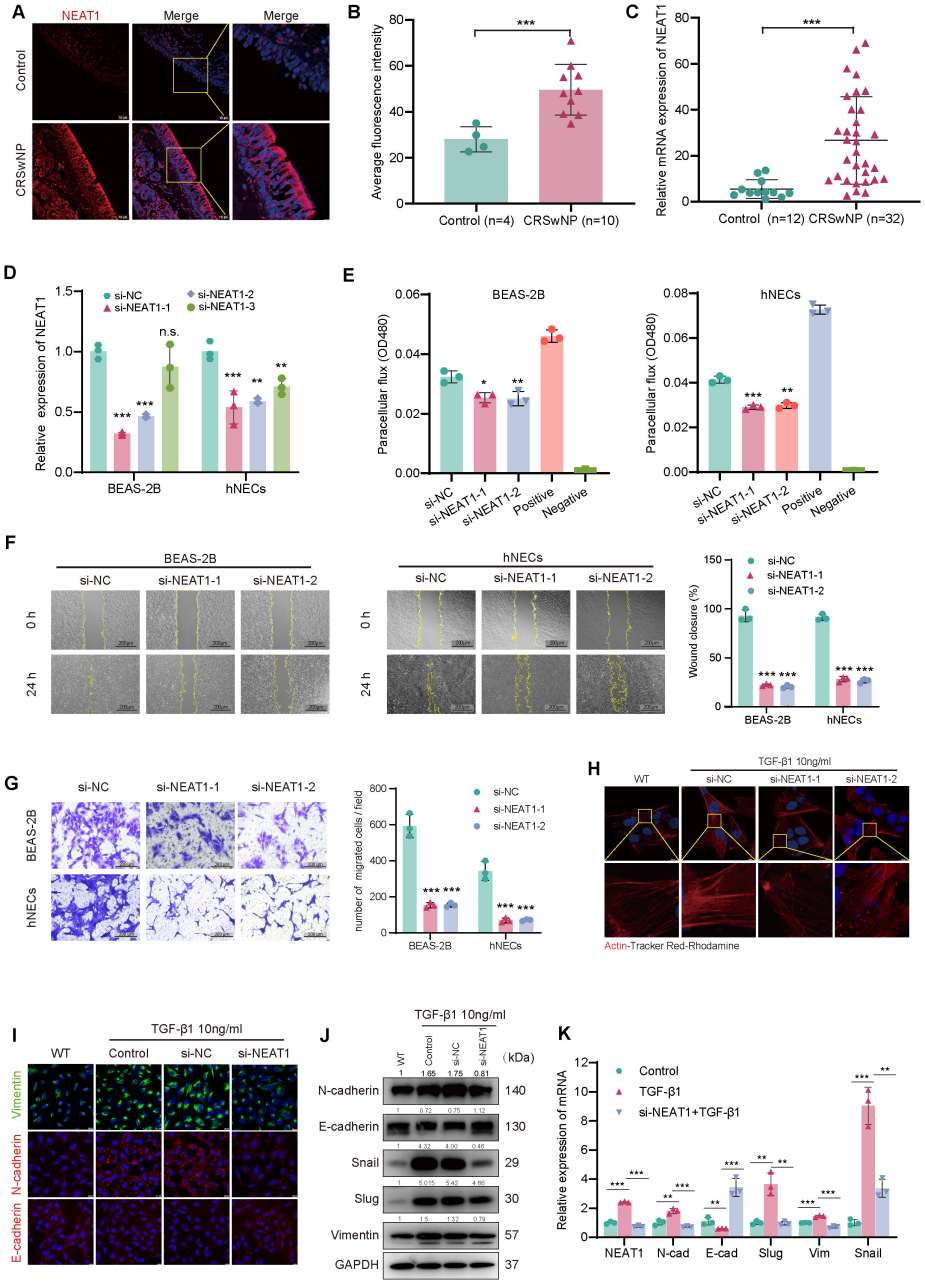
NEAT1 is upregulated in CRSwNP and promotes epithelial–mesenchymal transition (EMT). **(A)** FISH staining shows elevated NEAT1 expression (red) in CRSwNP tissues, primarily localized in the cytoplasm. Nuclei were counterstained with DAPI (blue). **(B)** Quantification of average fluorescence intensity in control (n = 4) and CRSwNP (n = 10) tissues. **(C)** qPCR analysis of NEAT1 expression in nasal tissues from control (n = 12) and CRSwNP patients (n = 32). **(D)** Validation of NEAT1 knockdown in BEAS-2B and hNECs after siRNA transfection. **(E)** FITC-dextran assay showing improved epithelial barrier function after NEAT1 knockdown. **(F)** Wound healing assay demonstrating reduced migration upon NEAT1 knockdown at 48 h **(G)** Transwell migration assay confirming reduced cell migration in both BEAS-2B and hNECs. **(H)** Phalloidin staining (Actin-Tracker Red-Rhodamine) showing reduced TGF-β1-induced stress fiber formation after NEAT1 knockdown. **(I)** Immunofluorescence staining of E-cadherin (red) and vimentin (green) in BEAS-2B cells. **(J)** Western blot analysis of EMT markers in control, TGF-β1-treated, and NEAT1 knockdown cells. **(K)** qPCR analysis of EMT-related genes after indicated treatments. Data are shown as mean ± SD; * p < 0.05, **p < 0.01, ***p < 0.001. n.s., not significant.

Cell transfection was performed using Lipofectamine 3000 (Invitrogen, USA). Before the official experiments, a total of 3 siRNAs were applied to perform knockdown, among which the one with the best knockdown efficiency was selected to perform the following functional experiments (**Figure 1D**). For miRNA modulation, miR-199-3p mimics, inhibitors, or corresponding negative controls were transfected to upregulate or suppress endogenous miRNA expression. For overexpression studies, PAK4 expression plasmids or empty vectors were transfected into cells. After 24–48 hours of transfection, cells were harvested for subsequent assays including qPCR, Western blot, or functional analyses. All oligonucleotides and plasmid constructs were obtained from GenePharma. The corresponding sequences are provided in **Supplementary Table 4**.

### 2.10 Dual-luciferase reporter assay

The interaction between miR-199-3p and its predicted targets NEAT1 and PAK4 was examined using a dual-luciferase reporter assay. The wild-type (WT) and mutant (MUT) 3′-UTR sequences of NEAT1 and PAK4 were cloned into the pmirGLO dual-luciferase vector, which expresses both firefly and Renilla luciferase. HEK293T cells were seeded in 24-well plates (1.5 × 10^2^ cells/well) and cultured overnight to reach ~70% confluence. Cells were co-transfected with 200 ng of reporter plasmid and 50 nM of miR-199-3p mimic or negative control (miR-NC) using Lipofectamine 3000 (Invitrogen) according to the manufacturer’s instructions. To ensure transfection efficiency and account for experimental variability, Renilla luciferase activity (expressed from the same pmirGLO vector) was used as an internal control. Cells were incubated for 48 hours post-transfection under standard culture conditions (37 °C, 5% CO_2_). Luciferase activity was measured using the Dual-Luciferase^®^ Reporter Assay System (Promega) on a microplate luminometer (Promega GloMax). Firefly luciferase signals were normalized to Renilla luciferase activity, and results were presented as relative luciferase activity (firefly/Renilla).

### 2.11 Transcriptome sequencing and analysis

BEAS-2B cells were transfected with si-NEAT1 or si-NC and harvested after 48 h. RNA from three replicates was extracted for sequencing, while one was used to confirm knockdown efficiency by qPCR. RNA-Seq was conducted by BGI Genomics using the DNBSEQ platform (SE50 mode). DEGs were identified using DESeq2 (|log_2_FC| ≥ 0.4, adjusted p < 0.05). Functional enrichment analyses, including GO and KEGG pathway analysis, were performed using clusterProfiler. Candidate downstream targets of NEAT1 were selected based on expression trends and functional relevance, with PAK4 identified for further validation.

### 2.12 Murine nasal polyp model

Twelve SPF-grade female BALB/c mice (4 weeks old, 18–22 g) were randomly divided into control and model groups (n = 6). To induce nasal polyps, mice in the model group received intraperitoneal OVA (25 μg) with aluminum hydroxide (2 mg) on days 0 and 5, followed by intranasal 3% OVA alone or with SEB (10 ng) for 90 days. Controls received PBS on the same schedule. Mice were euthanized 24 h after the final exposure, and nasal mucosa was collected, fixed in 4% paraformaldehyde, paraffin-embedded, and sectioned (4 μm) for subsequent analysis(Approval No. IACUC-CQMU-2023-0249).

### 2.13 Hematoxylin and eosin staining

Paraffin-embedded nasal sections (4 μm) were deparaffinized, rehydrated, and stained with hematoxylin and eosin to visualize cellular and tissue structures. After dehydration and mounting, slides were examined under a light microscope to assess histology and inflammation.

### 2.14 Immunohistochemistry

Paraffin-embedded nasal sections (4 μm) were deparaffinized, rehydrated, and subjected to antigen retrieval in citrate buffer (pH 6.0). After blocking with 3% hydrogen peroxide, sections were incubated overnight at 4 °C with primary antibodies, followed by HRP-conjugated secondary antibodies. DAB was used for visualization, and nuclei were counterstained with hematoxylin. Slides were dehydrated, mounted, and examined microscopically.

### 2.15 Fluorescence *in situ* hybridization

#### 2.15.1 Cell climbing slides

RNA-FISH was performed to determine the subcellular localization of NEAT1 in nasal epithelial cells. A Cy3-labeled probe specifically targeting NEAT1 was synthesized by GenePharma (shanghai). The BEAS-2B cells and primary hNECs were seeded on sterile glass coverslips and cultured to 60–70% confluence. Cells were fixed in 4% paraformaldehyde (PFA) for 15 minutes at room temperature, followed by permeabilization with 0.5% Triton X-100 in PBS for 10 minutes. Pre-hybridization was performed at 42 °C for 30 minutes in hybridization buffer Hybridization with the Cy3-labeled NEAT1 probe was carried out overnight (12–18 h) at 37 °C in a humidified chamber. After hybridization, cells were washed sequentially with 0.1% Buffer F,2× Buffer C, 1× Buffer C at 42 °C to reduce background. Nuclei were counterstained with DAPI for 5 minutes. Coverslips were mounted with antifade mounting medium and imaged using Scanning Confocal Microscope equipped with DAPI and Cy3 filter sets. Images were acquired and processed using ImageJ software. FISH sequences are listed in **Supplementary Table 5**.

#### 2.15.2 Tissue samples

The FISH probe sequences for mice differ from those for humans, therefore new probes were specifically redesigned for this study, with the detailed probe sequences provided in the (**Supplementary Table 5**). The paraffin sections were first dewaxed and cleared in xylene, followed by hydration through a graded alcohol series (100%, 95%, 90%, 80%, and 70%) with 10-minute incubations at each concentration, then washed twice with PBS. Subsequently, the sections were digested with proteinase K for 20 minutes. Pre-denaturation was performed using 200 μL of pre-heated (78°C) solution per well for 8 minutes, followed by dehydration through a reverse alcohol gradient (70% to 100%). Denatured probes (pre-treated at 73°C for 5 minutes) were applied to each section, and hybridization was carried out at 37°C for 12–16 hours overnight. The following day’s procedures were identical to those described for the cell climbing sections.

#### 2.15.3 Nuclear and cytoplasmic RNA isolation assay

BEAS-2B cells wase stimulated by TGF-β1 for 48 hours, after which cell precipitate was collected and utilized for extracting RNA in nuclei and cytoplasm using Nuclear and Cytoplasmic Extraction Kit (E101)+FreeZol Reagent(R711)(purchased from Novizan) according to manufacturer’s protocol, followed by reverse transcription and qPCR to examine the expression levels of NEAT1, with U6 and GAPDH as the internal reference for nucleus and cytoplasm.

### 2.16 Immunofluorescence staining

Cells grown on glass coverslips were fixed with 4% paraformaldehyde and permeabilized with 0.1% Triton X-100. After blocking with 5% BSA, samples were incubated overnight at 4°C with primary antibodies, followed by fluorophore-conjugated secondary antibodies (Alexa Fluor 488 or 594) for 1 h in the dark. Nuclei were stained with DAPI, and coverslips were mounted with anti-fade medium. Images were captured using a fluorescence microscope.

### 2.17 Bioinformatics prediction of RNA interactions

Potential miRNAs interacting with lncRNA NEAT1 were predicted using the ENCORI database (https://rnasysu.com/encori/) (25), which integrates CLIP-Seq-supported RNA–RNA interaction data. Candidate miRNAs targeting PAK4 were identified using TargetScanHuman (https://www.targetscan.org/vert_72/) (26) and miRDB (https://mirdb.org/) (27). Overlapping miRNAs predicted by multiple databases were selected for further validation in subsequent functional assays.

### 2.18 Statistical analysis

Data are presented as mean ± standard deviation (SD). Statistical analyses were conducted using GraphPad Prism 9.0. Comparisons between two groups were made using unpaired Student’s *t*-test. Pearson correlation analysis was used to assess the linear relationship between the expression levels of NEAT1, miR-199-3p, and PAK4. A *p*-value < 0.05 was considered statistically significant.

## 3 Results

### 3.1 NEAT1 is upregulated in nasal polyps and promotes EMT in nasal epithelial cells

To evaluate NEAT1 expression in CRSwNP, fluorescence *in situ* hybridization (FISH) was performed on nasal mucosal tissues from CRSwNP patients and healthy controls. NEAT1 expression was markedly increased in CRSwNP epithelial cells and predominantly localized in the cytoplasm (**Figures 1A, B**), suggesting a potential post-transcriptional regulatory role. This finding was further confirmed by qPCR analysis in an expanded sample set (n = 32 CRSwNP, n = 12 control), which showed significantly higher NEAT1 expression in the CRSwNP group (**Figure 1C**). To investigate the biological function of NEAT1, siRNA-mediated knockdown was performed in BEAS-2B cells and hNECs qPCR confirmed efficient suppression of NEAT1 expression. Before the official experiments, a total of 3 siRNAs were applied to perform knockdown, among which the one with the best knockdown efficiency was selected to perform the following functional experiments. To ease the concern of off-target issues, here we displayed the experimental results obtained by using the other two siRNA (**Figures 1F, G**). Functional assays revealed that NEAT1 knockdown significantly enhanced epithelial barrier integrity, as evidenced by reduced FITC-dextran permeability in both BEAS-2B and hNECs (**Figure 1E**). Moreover, wound healing (**Figure 1F**) and Transwell migration assays (**Figure 1G**) demonstrated a marked decrease in cell motility following NEAT1 knockdown.

Since epithelial-mesenchymal transition (EMT) is closely linked to cytoskeletal remodeling and enhanced migration, we evaluated F-actin distribution using Actin-Tracker Red-Rhodamine staining after TGF-β1 stimulation. TGF-β1 induced prominent stress fiber formation and spindle-shaped morphology, indicative of EMT, whereas NEAT1 knockdown attenuated these changes, restoring a cortical actin distribution and epithelial morphology (**Figure 1H**).

To further confirm the effect of NEAT1 on EMT at the molecular level, immunofluorescence, Western blot, and qPCR analyses were performed. Knockdown of NEAT1 reversed TGF-β1-induced EMT marker expression, as evidenced by increased E-cadherin protein levels and decreased N-cadherin, vimentin, Snail, and Slug at both mRNA and protein levels (**Figures 1I–K**).

### 3.2 NEAT1 positively regulates PAK4 via inflammatory cues and is upregulated in a murine nasal polyp model

**Figure 2:**
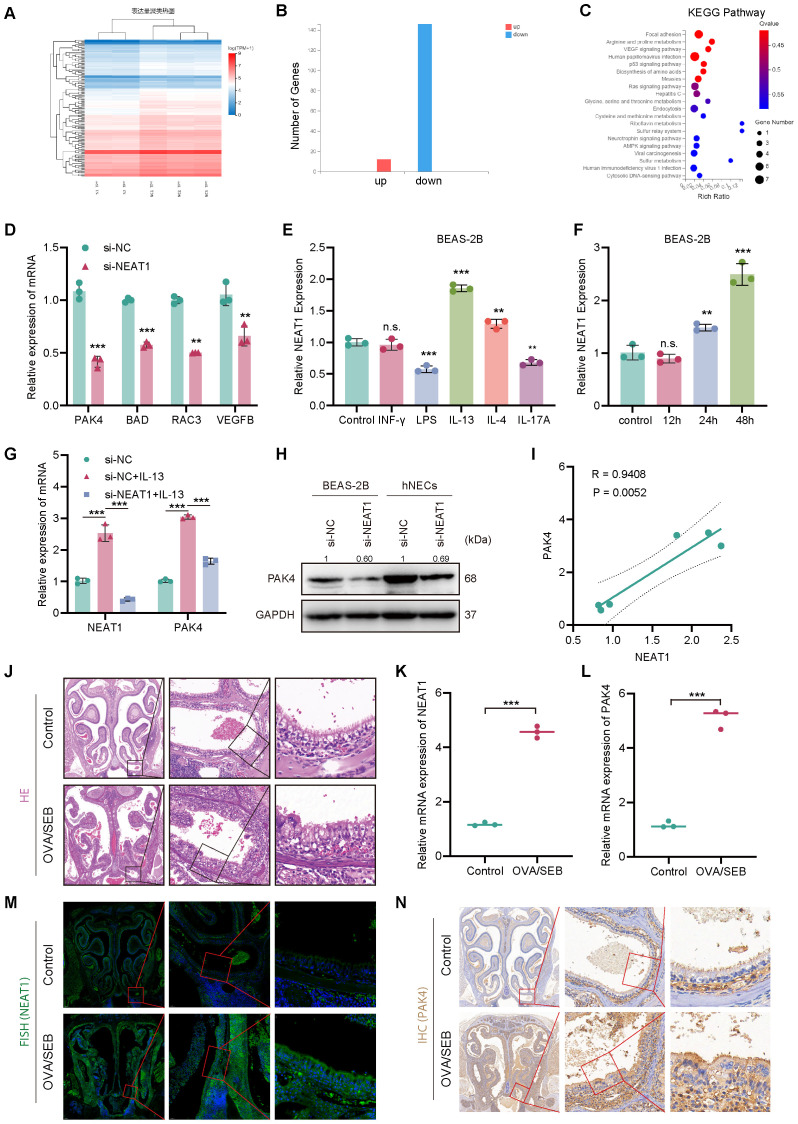
NEAT1 regulates PAK4 expression via inflammatory stimuli and is upregulated *in vivo*. **(A)** Heatmap of differentially expressed genes in BEAS-2B cells after NEAT1 knockdown; si-NEAT1–3 was excluded as an outlier. **(B)** Bar graph showing the number of upregulated and downregulated genes. **(C)** KEGG enrichment analysis highlighting significant enrichment in the focal adhesion pathway. **(D)** qPCR validation of selected downregulated genes after NEAT1 knockdown. **(E, F)** qPCR analysis of NEAT1 expression in BEAS-2B cells after stimulation with inflammatory cytokines **(E)** and time-course of IL-13 treatment **(F)**. **(G)** NEAT1 and PAK4 expression following IL-13 stimulation and/or NEAT1 knockdown. **(H)** Western blot showing PAK4 protein levels in BEAS-2B and hNECs. **(I)** Pearson correlation analysis of NEAT1 and PAK4 mRNA levels under IL-13 stimulation. **(J)** HE staining of nasal mucosa from control and OVA/SEB-induced polyp model mice. **(K, L)** qPCR analysis of Neat1 and PAK4 mRNA levels in mouse nasal tissue. **(M)** FISH detection of Neat1 expression in control and model mice. **(N)** IHC staining of PAK4 in nasal tissues. Data are presented as mean ± SD; **p < 0.01, ***p < 0.001. n.s., not significant.

To explore the downstream mechanisms of NEAT1 in epithelial–mesenchymal transition (EMT), transcriptome sequencing was performed in BEAS-2B cells following NEAT1 knockdown. After outlier exclusion, differential gene expression analysis revealed predominant downregulation of genes and significant enrichment in the focal adhesion pathway, which is closely associated with cytoskeletal remodeling and cell–matrix interactions (**Figures 2A–C**). Among the downregulated genes, PAK4 showed the most pronounced decrease, as validated by qPCR (**Figure 2D**), suggesting its potential role as a key mediator of NEAT1-driven EMT. In addition to PAK4, qPCR validation of selected genes from the enriched KEGG pathways confirmed significant downregulation of LAMA5,PPP1R12C,following NEAT1 knockdown (**Supplementary Materials**). These genes are involved in focal adhesion and cytoskeletal regulation, further supporting a broader role for NEAT1 in epithelial remodeling.

To investigate regulatory cues affecting NEAT1, BEAS-2B cells were treated with inflammatory stimuli representing different CRSwNP endotypes. IL-13, a Th2 cytokine central to eosinophilic nasal polyps (ENP), significantly upregulated NEAT1 expression in a time-dependent manner (**Figures 2E, F**). Furthermore, IL-13 stimulation increased both NEAT1 and PAK4 expression, while NEAT1 knockdown suppressed IL-13-induced PAK4 upregulation at both mRNA and protein levels (**Figures 2G, H**). Pearson correlation analysis confirmed a strong positive correlation between NEAT1 and PAK4 expression under IL-13 stimulation (R = 0.94, p = 0.0052; **Figure 2I**).

To validate these findings *in vivo*, a murine nasal polyp model was established using OVA/SEB sensitization. HE staining confirmed characteristic polypoid lesions (**Figure 2J**). qPCR and FISH analyses showed significant upregulation of NEAT1 in nasal tissues from model mice (**Figures 2K, M**), while IHC and qPCR revealed concurrent overexpression of PAK4 (**Figures 2L, N**). Additionally, immunohistochemical analysis of clinical samples revealed that PAK4 expression was higher in nasal polyp tissues compared to normal nasal mucosa (**Supplementary Figure 1**). These results support a functional NEAT1–PAK4 axis in CRSwNP pathogenesis, particularly in Th2-driven ENP.

### 3.3 PAK4 overexpression reverses the inhibitory effects of NEAT1 knockdown on EMT, cytoskeletal remodeling, and cell migration

**Figure 3:**
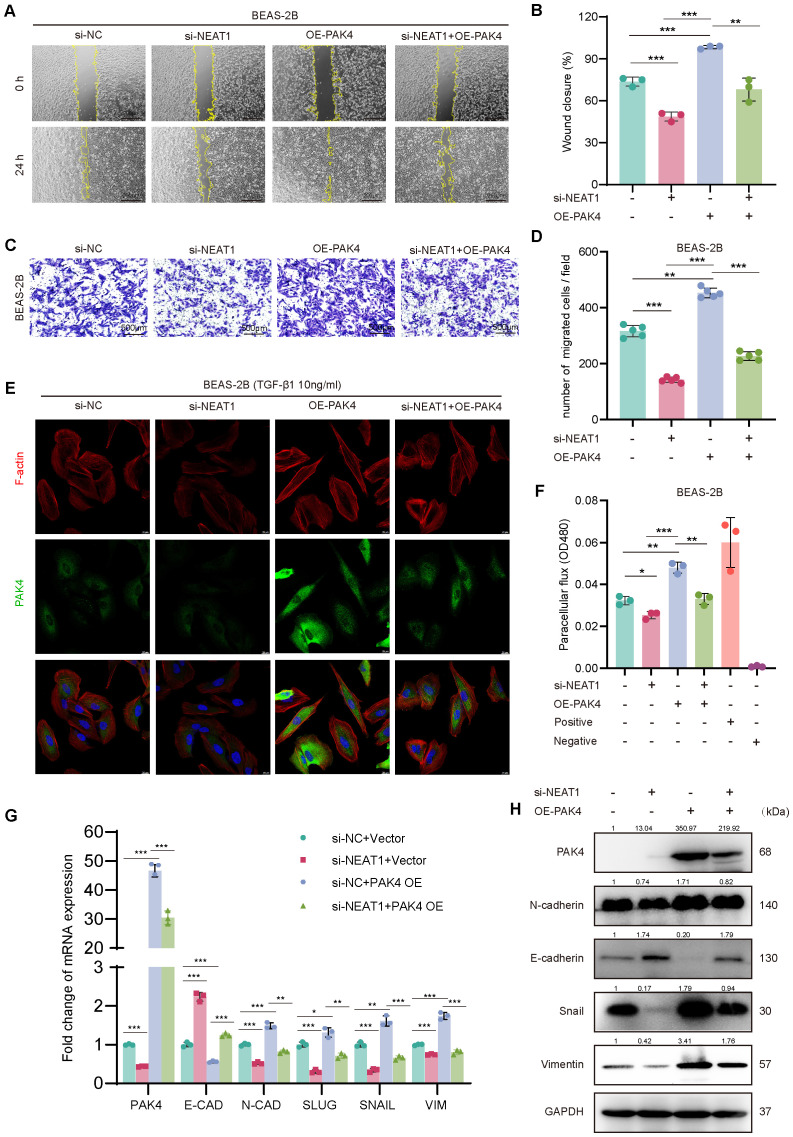
PAK4 overexpression reverses the inhibitory effects of NEAT1 knockdown on epithelial cell migration and EMT. **(A)** Wound healing assay of BEAS-2B cells under different treatment conditions (si-NC, si-NEAT1, OE-PAK4, si-NEAT1 + OE-PAK4) at 0 h and 24 h **(B)** Quantification of wound closure percentage. **(C)** Transwell assay showing migrated BEAS-2B cells under the same treatments. **(D)** Quantification of migrated cell numbers per field. **(E)** Immunofluorescence staining of F-actin (red), PAK4 (green), and nuclei (DAPI, blue) after TGF-β1 (10 ng/mL) stimulation. **(F)** Paracellular flux (FITC-dextran permeability assay) indicating epithelial barrier integrity. **(G)** qPCR analysis of EMT-related gene expression under different treatment groups. **(H)** Western blot analysis of PAK4, E-cadherin, N-cadherin, Snail, and Vimentin protein levels. GAPDH served as a loading control. Data are presented as mean ± SD; *p < 0.05, **p < 0.01, ***p < 0.001.

To assess whether PAK4 mediates the effects of NEAT1 on epithelial dynamics, we overexpressed PAK4 in BEAS-2B cells with NEAT1 knockdown. Wound healing and Transwell migration assays showed that PAK4 overexpression significantly restored the reduced migration capacity caused by NEAT1 silencing, as indicated by increased wound closure and a higher number of migrated cells (**Figures 3A–D**).

Since NEAT1 knockdown was previously shown to inhibit cytoskeletal remodeling in response to TGF-β1, we examined whether PAK4 overexpression could reverse this effect. Phalloidin staining revealed that while NEAT1 knockdown suppressed stress fiber formation and promoted cortical actin distribution, PAK4 overexpression restored cytoplasmic stress fiber assembly and a mesenchymal-like morphology (**Figure 3E**). Furthermore, PAK4 overexpression attenuated the barrier-protective effect of NEAT1 knockdown, as shown by increased FITC-dextran permeability (**Figure 3F**).

At the molecular level, qPCR and Western blot analyses demonstrated that PAK4 overexpression reversed NEAT1 knockdown-induced changes in EMT markers. Specifically, E-cadherin expression decreased, while N-cadherin, Vimentin, Snail, and Slug levels increased, consistent with EMT activation (**Figures 3G, H**). These findings support that PAK4 acts downstream of NEAT1 to promote EMT, cytoskeletal reorganization, and cell motility in nasal epithelial cells.

### 3.4 NEAT1 functions as a ceRNA to upregulate PAK4 by sponging miR-199-3p in nasal epithelial cells

**Figure 4:**
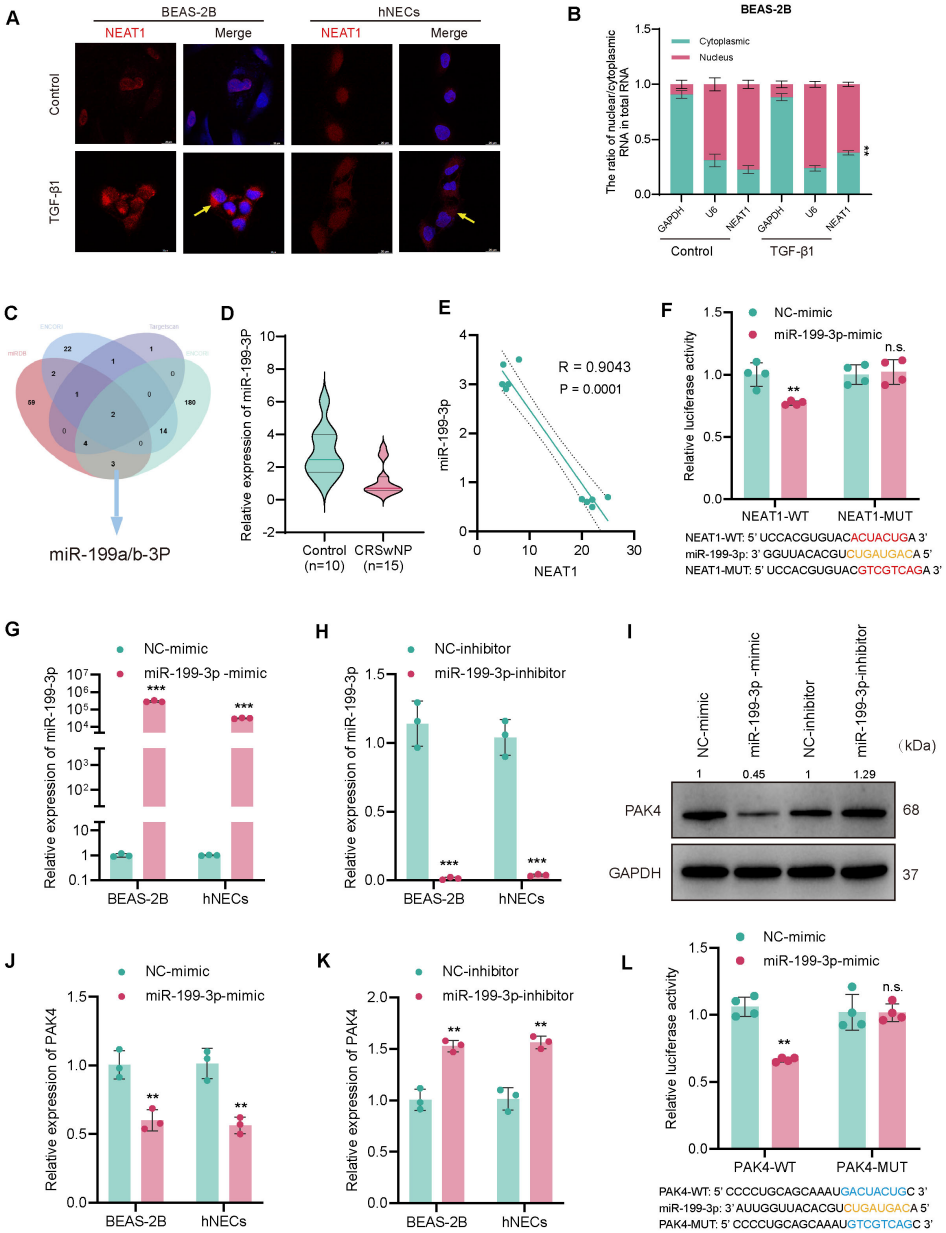
NEAT1 sponges miR-199-3p to regulate PAK4 via a ceRNA mechanism. **(A)** RNA-FISH showing NEAT1 localization (red) in BEAS-2B and hNECs, with nuclei counterstained by DAPI (blue); TGF-β1 stimulation increased NEAT1 expression. **(B)** QPCR analysis of NEAT1 distribution in nuclear and cytoplasmic RNA fractions. **(C)** Venn diagram integrating ENCORI, TargetScan, and miRDB predicting miR-199-3p as a common target of NEAT1 and PAK4. **(D)** Violin plot of miR-199-3p expression in CRSwNP (n = 15) *vs*. control (n = 10) tissues. **(E)** Pearson correlation analysis showing inverse relationship between NEAT1 and miR-199-3p expression. **(F)** Dual-luciferase assay validating direct binding of miR-199-3p to NEAT1-WT but not NEAT1-MUT. **(G, H)** qPCR validation of miR-199-3p overexpression and knockdown in BEAS-2B and hNECs. **(J, K)** qPCR showing miR-199-3p mimic decreased PAK4 expression, while inhibitor increased it. **(I)** Western blot confirming miR-199-3p regulation of PAK4 protein levels. **(L)** Luciferase assay showing miR-199-3p directly targets PAK4 3′UTR; no effect seen in mutant construct. Data are shown as mean ± SD; *p < 0.05, **p < 0.01, ***p < 0.001, n.s., not significant.

Given NEAT1’s cytoplasmic localization and its role in upregulating PAK4, we hypothesized that NEAT1 may act as a competing endogenous RNA (ceRNA) by binding and suppressing miR-199-3p. RNA-FISH revealed that NEAT1 was predominantly localized in the cytoplasm of BEAS-2B and hNECs, and its expression was significantly induced by TGF-β1 stimulation (**Figure 4A**). In addition, to further confirm the authenticity of the cytoplasmic-enrichment of NEAT1, qPCR was performed on the nuclear/cytoplasmic RNA extracts in BEAS-2B cells before/after TGF-β1(10 ng/ml 48h) stimulation, and the results indicated that NEAT1 was originally localized in nuclei, but the expression of NEAT1 in cytoplasm at transcriptional level was increased after TGFb1 stimulation, and the difference in nucleus/cytoplasm ratio of NEAT1 levels before/after stimulation was statistically significant (p = 0.003) (**Figure 4B**). Integrated miRNA target prediction using ENCORI, TargetScan, and miRDB identified miR-199a/b-3p as a shared miRNA candidate predicted to bind both NEAT1 and PAK4 (**Figure 4C**). Given that miR-199a-3p and miR-199b-3p share identical mature sequences, we referred to this functional miRNA as miR-199-3p in subsequent experiments. qPCR analysis confirmed that miR-199-3p was significantly downregulated in CRSwNP tissues compared to controls (**Figure 4D**), and Pearson correlation analysis showed a strong inverse correlation between NEAT1 and miR-199-3p expression (R = −0.9043, p = 0.0001) (**Figure 4E**).

Dual-luciferase assays validated the direct interaction between NEAT1 and miR-199-3p. Co-transfection of miR-199-3p mimic significantly suppressed luciferase activity in cells carrying the NEAT1-WT reporter but not the mutant (NEAT1-MUT), confirming direct binding (**Figure 4F**). Transfection of miR-199-3p mimic or inhibitor successfully modulated miR-199-3p expression in BEAS-2B and hNECs (**Figures 4G, H**), and inversely regulated PAK4 expression at both the mRNA and protein levels (**Figures 4H–J**). Finally, miR-199-3p mimic significantly reduced luciferase activity of the PAK4-WT 3′UTR reporter, but not the mutant construct (PAK4-MUT), confirming PAK4 as a direct downstream target of miR-199-3p (**Figure 4L**).

These results demonstrate that NEAT1 regulates PAK4 by acting as a ceRNA to sponge miR-199-3p, thereby promoting EMT and contributing to CRSwNP pathogenesis.

### 3.5 Rescue experiments validate the functional integrity of the NEAT1–miR-199-3p–PAK4 axis

**Figure 5:**
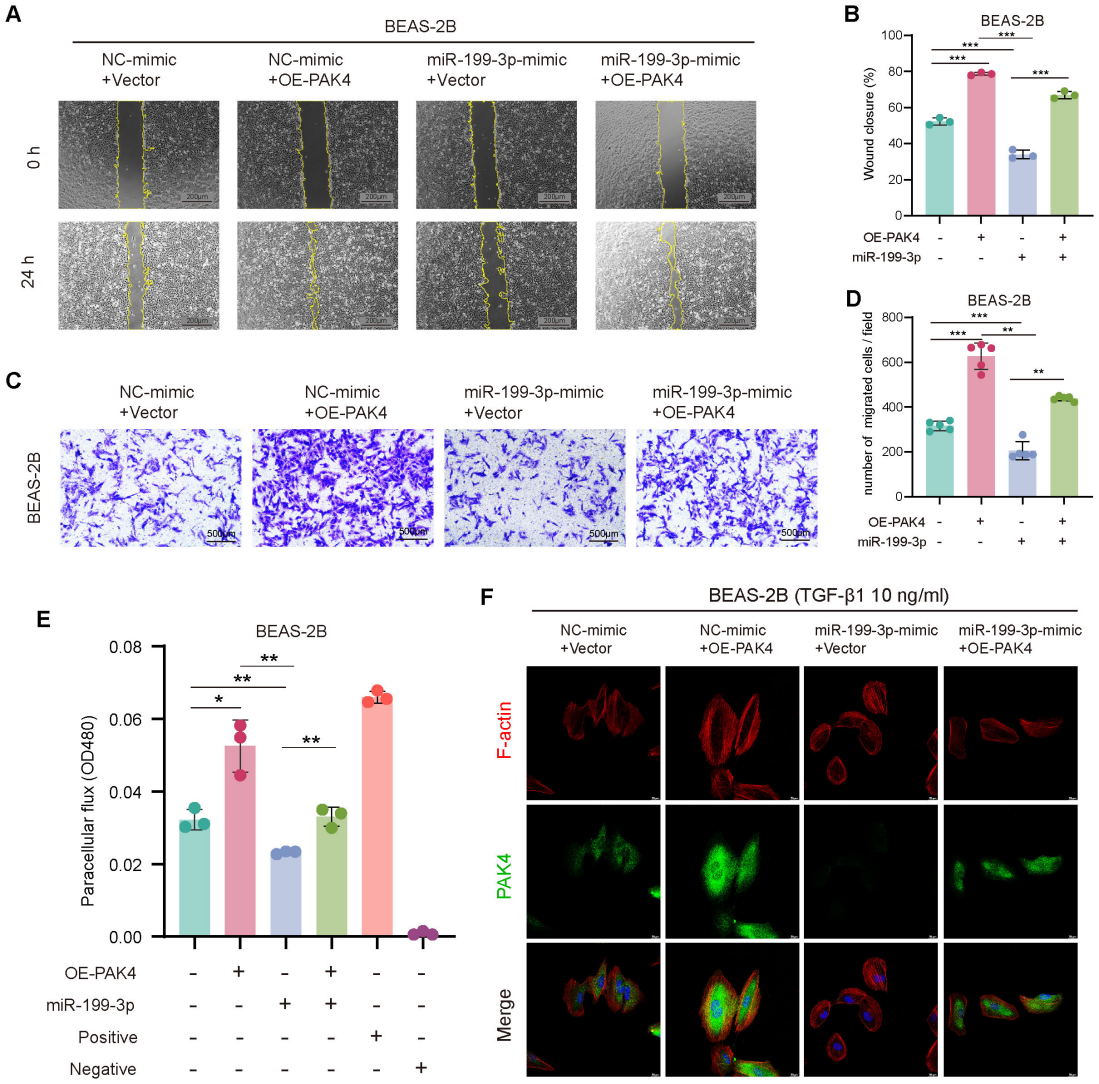
miR-199-3p reverses the pro-EMT effects of PAK4 overexpression in BEAS-2B cells. **(A)** Wound healing assay at 0 h and 24 h showing enhanced cell migration with PAK4 overexpression, which was reversed by co-transfection of miR-199-3p mimic. **(B)** Quantification of wound closure percentage. **(C)** Transwell migration assay showing increased migration with PAK4-OE, suppressed by miR-199-3p. **(D)** Quantification of migrated cells per field. **(E)** FITC-dextran permeability assay showing PAK4-induced barrier dysfunction and its reversal by miR-199-3p mimic. **(F)** Phalloidin staining (F-actin in red, PAK4 in green, nuclei in blue) revealing that PAK4 induces cytoskeletal stress fiber formation, which is reduced by miR-199-3p mimic. Data are shown as mean ± SD; *p < 0.05, **p < 0.01, ***p < 0.001.

To assess the functional role of miR-199-3p in counteracting PAK4-mediated EMT, we performed a series of rescue assays in BEAS-2B cells co-transfected with PAK4 overexpression vector and miR-199-3p mimic. Wound healing and Transwell assays demonstrated that PAK4 overexpression significantly enhanced the migratory capacity of BEAS-2B cells, as evidenced by increased wound closure rate and the number of migrated cells. However, co-transfection with miR-199-3p mimic effectively suppressed this enhancement, reducing both wound healing and migration to near baseline levels (**Figures 5A–D**). These findings suggest that miR-199-3p mitigates the pro-migratory effects induced by PAK4. To evaluate epithelial barrier function, we conducted a FITC-dextran permeability assay. PAK4 overexpression markedly increased paracellular dextran flux, indicating compromised barrier integrity. This effect was significantly reversed by co-expression of miR-199-3p, which restored barrier function to levels comparable to controls (**Figure 5E**). Phalloidin staining was used to assess actin cytoskeletal changes associated with EMT. Cells overexpressing PAK4 exhibited enhanced stress fiber formation and increased cytoplasmic F-actin accumulation, indicative of cytoskeletal remodeling. Notably, miR-199-3p mimic co-expression reversed these changes, leading to reduced stress fiber density and a return to cortical actin distribution, consistent with an epithelial phenotype (**Figure 5F**). these results demonstrate that miR-199-3p can effectively reverse the pro-EMT phenotypes induced by PAK4, including enhanced migration, impaired barrier function, and cytoskeletal reorganization.

**Figure 6:**
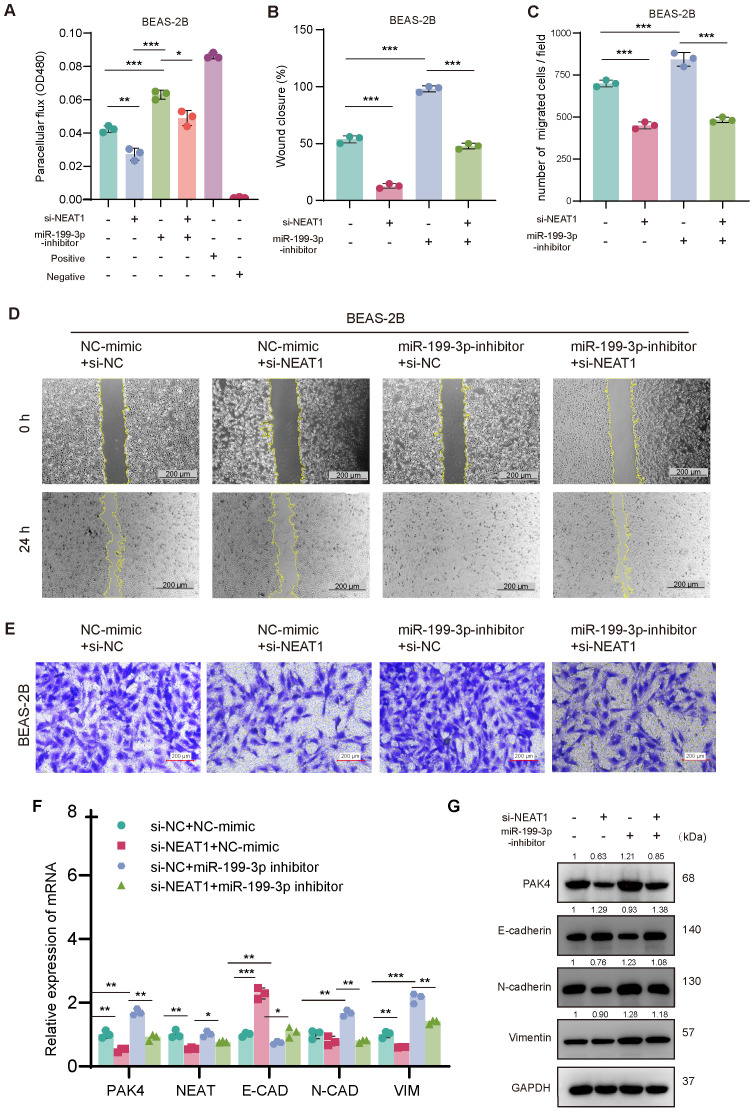
NEAT1 regulates EMT through the miR-199-3p/PAK4 axis in BEAS-2B cells. **(A)** FITC-dextran assay confirms that NEAT1 knockdown improves barrier function, which is disrupted by miR-199-3p inhibition. **(B–E)** Wound healing and Transwell migration assays show that miR-199-3p inhibition reverses NEAT1 knockdown–induced suppression of migration, while subsequent NEAT1 silencing rescues the migratory phenotype. **(F, G)** qPCR and Western blot analysis show that NEAT1 knockdown upregulates E-cadherin and suppresses PAK4, N-cadherin, Vimentin, Snail, and Slug; miR-199-3p inhibitor reverses this trend. Data are shown as mean ± SD; *p < 0.05, **p < 0.01, ***p < 0.001.

Next, we examined whether miR-199-3p also mediates NEAT1-driven phenotypes. Knockdown of NEAT1 suppressed cell migration and barrier dysfunction, as shown by decreased wound healing, reduced Transwell migration, and lower FITC-dextran permeability. These effects were partially reversed by miR-199-3p inhibition, suggesting that miR-199-3p is required for NEAT1-mediated EMT activation (**Figures 6A–E**).

At the molecular level, qPCR and Western blot analyses confirmed that NEAT1 knockdown downregulated PAK4 and mesenchymal markers (N-cadherin, Vimentin, Snail, Slug), while upregulating E-cadherin, consistent with EMT suppression. These effects were reversed by miR-199-3p inhibition, which restored PAK4 expression and EMT marker levels (**Figures 6F, G**).

Together, these findings demonstrate that miR-199-3p is a critical intermediate in the NEAT1/PAK4 regulatory circuit, and highlight the NEAT1/miR-199-3p/PAK4 axis as a key modulator of EMT and epithelial barrier dysfunction in nasal epithelial cells.

## 4 Discussion

CRSwNP remains a challenging inflammatory disorder due to its high recurrence rate and limited response to conventional therapies (3). Increasing evidence has highlighted the role of epithelial dysfunction and epithelial–mesenchymal transition (EMT) in the pathogenesis of CRSwNP, particularly in eosinophilic-dominant endotypes (7, 8, 28–30). However, the underlying regulatory networks remain incompletely defined. In this study, we identified long non-coding RNA NEAT1 as a key driver of EMT and epithelial barrier impairment in CRSwNP and elucidated its functional mechanism through the miR-199-3p/PAK4 axis.

We first demonstrated that NEAT1 is significantly upregulated in both clinical CRSwNP specimens and an OVA/SEB-induced murine nasal polyp model. Functional *in vitro* experiments confirmed that NEAT1 promotes cell migration, disrupts epithelial barrier integrity, and facilitates cytoskeletal remodeling, all hallmark features of EMT. These findings are consistent with previous studies implicating NEAT1 in EMT and inflammatory remodeling in various contexts, such as pulmonary fibrosis and cancers (12, 19, 31–33). Our transcriptomic analysis identified PAK4 as a key downstream effector of NEAT1. PAK4 is a serine/threonine kinase involved in cytoskeletal regulation, focal adhesion, and cell motility. PAK4 has been shown to activate EMT by modulating several key signaling cascades, such as the PI3K/AKT, MAPK/ERK, and Wnt/β-catenin pathways (21, 34–36). These pathways converge on transcriptional regulators including Snail, Slug, and Twist, promoting their expression or stability. Although we did not directly investigate these upstream signals, our results are consistent with previous findings suggesting that PAK4 functions as a central mediator connecting extracellular cues to EMT transcriptional programming. We showed that PAK4 expression was positively regulated by NEAT1 at both the mRNA and protein levels, and PAK4 overexpression successfully rescued the EMT-suppressive effects induced by NEAT1 knockdown. These results establish PAK4 as an essential mediator of NEAT1-driven epithelial remodeling in CRSwNP.

Mechanistically, we confirmed that NEAT1 acts through a ceRNA (competing endogenous RNA) mechanism by sequestering miR-199-3p, a microRNA known to suppress EMT and inflammation (37, 38). Dual-luciferase assays and rescue experiments provided direct evidence that NEAT1 binds miR-199-3p, thereby preventing it from inhibiting PAK4 translation. Notably, miR-199-3p expression was significantly downregulated in CRSwNP tissues and inversely correlated with NEAT1 expression, suggesting a functional imbalance in this regulatory axis.

Rescue experiments further reinforced the physiological relevance of this axis: overexpression of miR-199-3p reversed the EMT-promoting effects of PAK4, while inhibition of miR-199-3p abrogated the suppressive effects of NEAT1 knockdown. Together, these findings outline a complete NEAT1–miR-199-3p–PAK4 signaling circuit that governs epithelial plasticity and dysfunction in CRSwNP.

In this study, NEAT1 is proved to be partially translocate from nucleus to cytoplasm of nasal epithelial cells under TGF-β1 stimulation by FISH analysis and serve as a competing endogenous RNA (ceRNA) to regulate miR-199-3p and PAK4. Previous studies have reported that NEAT1 is originally recognized as a nuclear-enriched lncRNA which participates in remodeling chromatin, regulating transcription, holding RNA and interacting with miRNA by acting as a nuclear scaffold in the formation of paraspeckles (39–41). Given that evidences in this study indicated its partial translocation to cytoplasm in nasal epithelial cells, we mainly focused on its cytoplastic functions, but the part remained in nucleus may still function as a role in the regulation of CRSwNP pathogenesis. In addition to the miR-199-3p/PAK4 axis, transcriptomic and qPCR data suggested that NEAT1 may influence other EMT-associated pathways, including focal adhesion and adherens junction signaling. This broader regulatory effect may act in concert with the PAK4 axis to promote cytoskeletal reorganization and epithelial plasticity.

In the broader context, our findings provide new insights into the molecular etiology of CRSwNP. NEAT1 serves as a converging point linking chronic inflammation (e.g., IL-13 signaling), epigenetic modulation, and structural remodeling through a miRNA-mediated axis. Given that IL-13 upregulated NEAT1 and PAK4 *in vitro*, the NEAT1/miR-199-3p/PAK4 axis may be particularly relevant in Th2-skewed eosinophilic nasal polyps, which are notoriously more resistant to conventional therapies (42, 43).

This study has several strengths, including the integration of transcriptomic profiling, *in vitro* and *in vivo* functional assays, and molecular interaction validation. However, certain limitations should be acknowledged. First, although we verified this axis in mouse models and patient tissues, clinical correlations with disease severity or recurrence rates were not deeply explored. Second, potential upstream regulators of NEAT1 remain unclear. Finally, while the NEAT1/miR-199-3p/PAK4 axis significantly influences EMT, additional downstream signaling components and feedback loops warrant further investigation.

**Figure 7:**
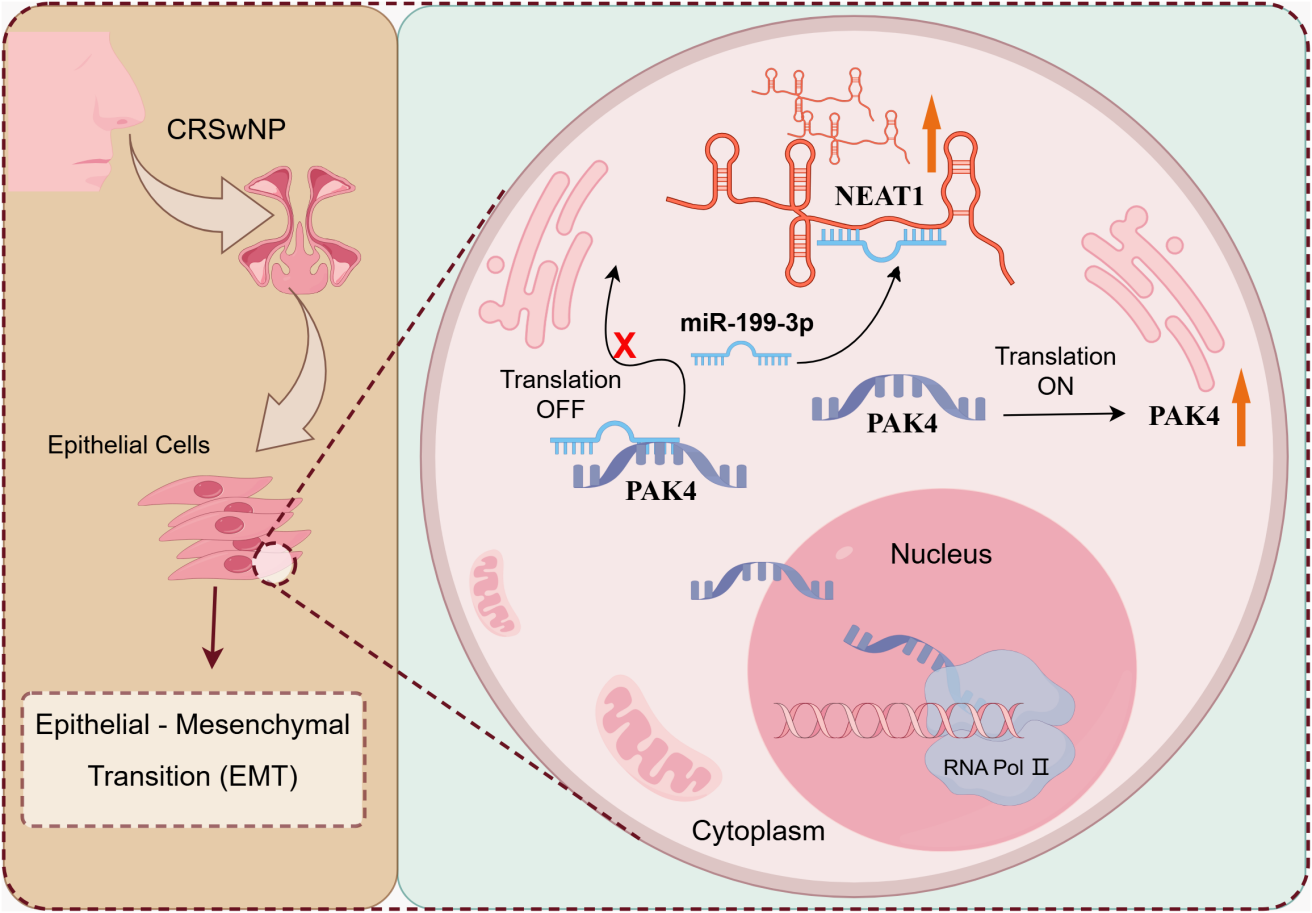
The mechanism diagram of NEAT1 regulating EMT changes in CRSwNP. LncRNA NEAT1 promotes epithelial-mesenchymal transformation of nasal polyp through the up-regulation of PAK4 axis by adsorption of miR-199-3.

In conclusion, we have identified NEAT1 as a critical lncRNA that promotes EMT and epithelial dysfunction in CRSwNP via a ceRNA mechanism targeting miR-199-3p and PAK4 (**Figure 7**). These findings provide a mechanistic framework for understanding nasal polyp formation and offer promising molecular targets for early diagnosis, endotype classification, and personalized therapy in CRSwNP.

## 5 Conclusion

In summary, this study demonstrates that the long non-coding RNA NEAT1 plays a pivotal role in promoting epithelial–mesenchymal transition (EMT) and epithelial barrier dysfunction in CRSwNP by acting as a competing endogenous RNA (ceRNA) to suppress miR-199-3p and upregulate PAK4. Functional and mechanistic evidence from transcriptomic analysis, *in vitro* experiments, and *in vivo* validation collectively support the existence of a regulatory NEAT1/miR-199-3p/PAK4 axis. These findings not only advance our understanding of the molecular pathogenesis of CRSwNP but also highlight NEAT1 as a potential biomarker and therapeutic target for epithelial remodeling in chronic sinonasal inflammation.

## Data Availability

The raw sequencing data have been uploaded to the Sequence Read Archive (SRA) database, and the following accession numbers have been assigned: SRR34103286, SRR34103287, SRR34103288, SRR34103289, SRR34103290, and SRR34103291. Readers can retrieve and download the relevant data from the NCBI SRA using these accession numbers.
