# Supplementary material for: LncRNA NEAT1 promotes epithelial–mesenchymal transition in nasal polyp cells via the miR-199-3p/PAK4 axis
Source: Front Immunol. 2025 Jun 30;16:1613179. doi: 10.3389/fimmu.2025.1613179 (PMC12256258; doi:10.3389/fimmu.2025.1613179)
Supplement: Supplementary file 1 [file Table1.docx]

**Supplementary Table 1**

| **Abbreviation** | **Full Term** |
| --- | --- |
| CRSwNP | Chronic Rhinosinusitis with Nasal Polyps |
| EMT | Epithelial–Mesenchymal Transition |
| lncRNA | Long Non-Coding RNA |
| NEAT1 | Nuclear Enriched Abundant Transcript 1 |
| miRNA | MicroRNA |
| ceRNA | Competing Endogenous RNA |
| PAK4 | p21-Activated Kinase 4 |
| hNECs | Human Nasal Epithelial Cells |
| qPCR | Quantitative Polymerase Chain Reaction |
| FISH | Fluorescence In Situ Hybridization |
| FITC | Fluorescein Isothiocyanate |
| siRNA | Small Interfering RNA |
| si-NEAT1 | Small Interfering RNA Targeting NEAT1 |
| si-NC | Small Interfering RNA Negative Control |
| OE | Overexpression |
| WT | Wild-Type |
| MUT | Mutant |
| DAPI | 4',6-Diamidino-2-Phenylindole |
| F-actin | Filamentous Actin |
| IHC | Immunohistochemistry |
| IF | Immunofluorescence |
| HE | Hematoxylin and Eosin |
| PBS | Phosphate-Buffered Saline |
| BSA | Bovine Serum Albumin |
| HRP | Horseradish Peroxidase |
| ECL | Enhanced Chemiluminescence |
| DAB | 3,3'-Diaminobenzidine |
| GAPDH | Glyceraldehyde 3-Phosphate Dehydrogenase |
| U6 | U6 Small Nuclear RNA (reference gene for miRNA) |
| IL-13 | Interleukin-13 |
| Th2 | T Helper Type 2 |
| ENP | Eosinophilic Nasal Polyps |
| OVA | Ovalbumin |
| SEB | Staphylococcus aureus Enterotoxin B |
| DEGs | Differentially Expressed Genes |
| KEGG | Kyoto Encyclopedia of Genes and Genomes |
| GO | Gene Ontology |
| RNA-Seq | RNA Sequencing |
| SD | Standard Deviation |
| ANOVA | Analysis of Variance |
| NC | Negative Control |

**Supplementary Table 2**

| **Gene Name** | **Primer Name** | **Sequence (5ʹ–3ʹ)** | **Product Size** |
| --- | --- | --- | --- |
| NEAT1 | NEAT1-F | AAACGCTGGGAGGGTACAAG | 71 bp |
|  | NEAT1-R | ATGCCCAAACTAGACCTGCC |  |
| E-cadherin | E-CAD-F | AGCCAACCCCAATACAAAA | 120 bp |
|  | E-CAD-R | GGCTGCTGGCAATCTC |  |
| Vimentin | VIM-F | GACGCCATCAACACCGAGTT | 150 bp |
|  | VIM-R | CTTTGGTCTCATTGCACGCT |  |
| N-cadherin | N-CAD-F | TCAGGCGTCTGTAGAGGCTT | 132 bp |
|  | N-CAD-R | ATGCACATCCTTCGATAAGACTG |  |
| SNAI1 | SNAIL-F | ACTGCAACAAGGACACAT | 200 bp |
|  | SNAIL-R | CATAGTTAGTCACACCTCGT |  |
| SNAI2 | SLUG-F | CGAACTGGACACACATACAGTG | 185 bp |
|  | SLUG-R | CTGAGGATCTCTGGTTGTGGT |  |
| miR-199-3p | miR-199-3p-F | CAGTAGTCTGCACATTGGTTA |  |
| U6 | U6-F | GGAACGATACAGAGAAGATTAGC | 45 |
|  | U6-R | TGGAACGCTTCACGAATTTGCG |  |
| GAPDH | GAPDH-F | GAAGGTGAAGGTCGGAGT | 226 |
|  | GAPDH-R | GAAGATGGTGATGGGATTTC |  |

**Supplementary Table 3**

| **Antibody Name** | **Application** | **Supplier** | **Catalog Number** |
| --- | --- | --- | --- |
| GAPDH | WB | Santa cruz | sc-477724 |
| E-cadherin | IF/WB | CST | #3195 |
| N-cadherin | IF/WB | CST | #13116 |
| Vimentin | IF/WB | CST | #5741 |
| Snail | IF/WB | CST | #3879 |
| Slug | IF/WB | CST | #9585 |

**Supplementary Table 4**

| **Name** | **Sequence (5ʹ–3ʹ)** |
| --- | --- |
| si-NEAT1#1 | GCTGTGGAGTGTATGAATT |
| si-NEAT1#2 | GCACAATGCATGAGTGTTT |
| si-NEAT1#3 | CCAAATAGGCTTACAGATA |
| miR-199-3P-mimic Sense | ACAGUAGUCUGCACAUUGGUUA |
| miR-199-3P-mimic Antisense | ACCAAUGUGCAGACUACUGUUU |
| miR-199-3P-inhibitor | UAACCAAUGUGCAGACUACUGU |

**Supplementary Table 5**

| **Probe（*Homo sapiens*）** | **Sequence (5ʹ–3ʹ)** |
| --- | --- |
| NEAT1-2-1 | CTGAATTCTATTATATTACCCACACC |
| NEAT1-2-2 | TTTAAATTGATACAGCCATGCAAG |
| NEAT1-2-3 | ATTAATATTTATGTACTCTTGGGGTG |
| NEAT1-2-4 | GAAACACAAAATTAAAAGGCAAC |
| NEAT1-2-5 | CATAAAGTTGCCAAGTAGTCCA |
| NEAT1-2-6 | TCGCAAGTATTATTTTCTAAGAGGA |
| NEAT1-2-7 | CTTTAGTATAAGCACAATGGCAT |
| NEAT1-2-8 | CAACGATAACTCTAATAACCCAA |
| NEAT1-2-9 | TGTTAGTGGCTATGTAGGGAA |
| NEAT1-2-10 | TAAATGAAGGCAAAGAATGTGG |
| NEAT1-2-11 | CATATCGTATCTGTAAGCCTA |
| NEAT1-2-12 | CACCATTTACATACAGGCATC |
| NEAT1-2-13 | TCAGAGAAATAAGCCAGTCAC |
| NEAT1-2-14 | CATAACTTTTAATTTGCACAAGATCA |
| NEAT1-2-15 | GAATTATTTAAAAGAGAGGAAAGGCA |
| NEAT1-2-16 | AATATCCTTAAGATGTAAATGGC |
| NEAT1-2-17 | CAATCCCCAACATTTATACCC |
| NEAT1-2-18 | TTCTGGCTATATACTCAAAAGAAC |

| **Neat1 Probe**  **(*Mus musculus*)** | **Sequence** |
| --- | --- |
| Probe 1 | 5'-TCCATGAGTGCTCTGGTGGA-3' |
| Probe 2 | 5'-GCTTCCATGTAGGCTGATGC-3' |
| Probe 3 | 5'-GGAAGACTGCAGGAGGTGTT-3' |
| Probe 4 | 5'-ATCTGTGAGGCTCCGTGATC-3' |
| Probe 5 | 5'-TGGCAATGACTGCGTGGCTA-3' |
| Probe 6 | 5'-AGGCTGTGACCATGTGGGAA-3' |
| Probe 7 | 5'-CTCCAGGTAGCCTGACCTGA-3' |
| Probe 8 | 5'-AGCTGGAATGGTGCTCAGGA-3' |
| Probe 9 | 5'-GTGACCGAGTCTCCCTTGCA-3' |
| Probe 10 | 5'-AACGGAGCAGTTGGTGACCT-3' |
| Probe 11 | 5'-TGCAGAGGCTGCTCCATGAA-3' |
| Probe 12 | 5'-AGGAGGTGGAGTGTGCCATT-3' |
| Probe 13 | 5'-GGAAGGCTGCTTGAGGATGC-3' |
| Probe 14 | 5'-TCTCCAGTGGTGCCACTGTA-3' |
| Probe 15 | 5'-CCAGTGGCTGTGATCTGAGA-3' |
| Probe 16 | 5'-GCTCAGGTAGGAGGTGCTGA-3' |
| Probe 17 | 5'-AGCTGTTGAGGACAGCGTGG-3' |
| Probe 18 | 5'-TCAGTGAGGCTGAGTGTGGA-3' |
| Probe 19 | 5'-GAGGCTGCTGAGGATGCTGA-3' |
| Probe 20 | 5'-CTGGAGGTAGGCTGCTGTGA-3' |

**Supplementary Figure. 1**


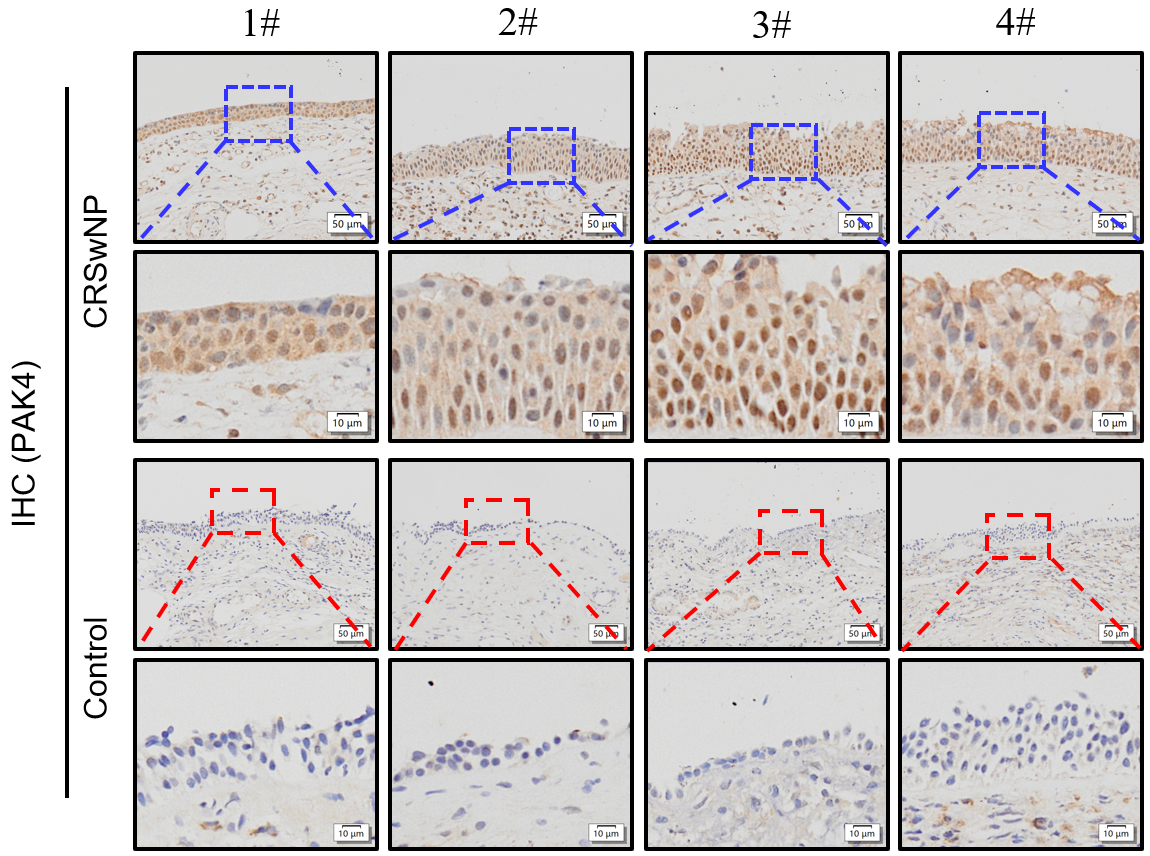


The IHC analysis showed that the expression level of PAK4 was significantly elevated in nasal polyp tissues of patients compared to the control group.
