# Supplementary material for: LncRNA NEAT1 promotes epithelial–mesenchymal transition in nasal polyp cells via the miR-199-3p/PAK4 axis
Source: Front Immunol. 2025 Jun 30;16:1613179. doi: 10.3389/fimmu.2025.1613179 (PMC12256258; doi:10.3389/fimmu.2025.1613179)
Supplement: Supplementary file 2 [file Table2.docx]

Supplementary materials

To further validate the transcriptomic findings, qPCR analyses of additional representative genes from these pathways were performed, including LAMA5, PPP1R12C, which showed consistent downregulation upon NEAT1 silencing (Supplementary materials).


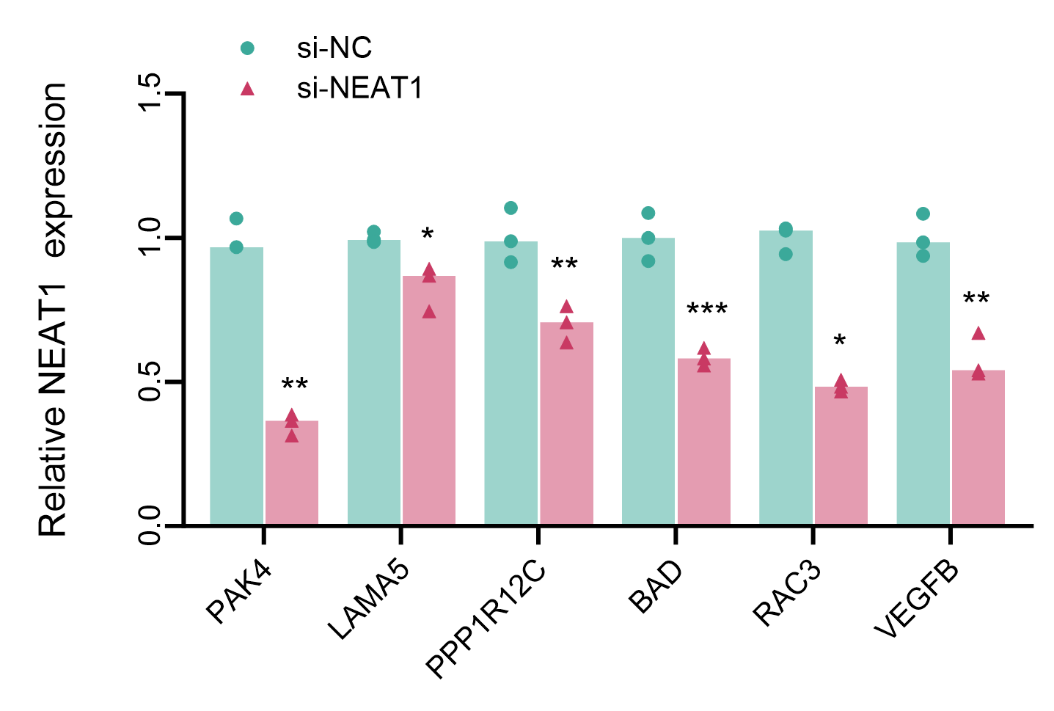


| **Gene Name** | **Primer Name** | **Sequence (5ʹ–3ʹ)** | **Product Size** |
| --- | --- | --- | --- |
| LAMA5 | LAMA5-F | CACCATCCCGCCCGACTG | 75 bp |
|  | LAMA5-R | CACTCCTCACAGCCGACCAG |  |
| PPP1R12C | PPP1R12C-F | CAACATCGCCGCCGTCAAC | 136 bp |
|  | PPP1R12C-R | TTCCTCTTCTGCCCGCTTGG |  |
